# Evaluation of the Novel CE-IVD-Marked Multiplex PCR QIAstat-Dx Meningitis/Encephalitis Panel

**DOI:** 10.1128/spectrum.05144-22

**Published:** 2023-04-12

**Authors:** Anu Humisto, Jenni Antikainen, Tanja Holma, Hanna Jarva, Anne Toivonen, Raisa Loginov, Laura Mannonen

**Affiliations:** a Department of Clinical Microbiology, Helsinki University Hospital and University of Helsinki, Helsinki, Finland; University of Chicago

**Keywords:** FilmArray, HSV-1, HSV-2, QIAstat, central nervous system infections, cerebrospinal fluid, CSF, encephalitis, meningitis, multiplex PCR

## Abstract

Central nervous system (CNS) infections such as meningitis and encephalitis are life-threatening conditions that demand hospital care and prompt identification of the causative agent. Since 2015, there has been only one CE-IVD-marked rapid multiplexed diagnostic assay in cassette format for bacterial and viral detection from cerebrospinal fluid (CSF): the BioFire FilmArray meningitis/encephalitis (ME) panel. In the beginning of 2022, Qiagen introduced the QIAstat-Dx meningitis/encephalitis panel. It is a CE-IVD-marked multiplex PCR cassette test intended for the identification of suspected infectious meningitis, encephalitis, or meningoencephalitis caused by bacterial, viral, or fungal pathogens. In this study, we evaluated patient and quality control samples using the QIAstat-Dx meningitis/encephalitis panel and compared the results to those of the BioFire FilmArray meningitis/encephalitis panel and reference methods (current routine analysis methods in our laboratory, PCR, or cultivation). The combined positive percent agreement between the two panel assays was 100%, and the negative percent agreement was 94%. We further compared specifically herpes simplex virus 1 (HSV-1), HSV-2, and varicella-zoster virus (VZV) dilution series using six commercial herpesvirus assays, including the two cassette tests. The results suggested that real-time PCR methods (with separate extraction) were the most sensitive methods. When comparing the cassette tests, the BioFire FilmArray meningitis/encephalitis panel produced more positive results than the QIAstat-Dx meningitis/encephalitis panel in the herpesvirus analyses.

**IMPORTANCE** The diagnosis of infectious meningitis and encephalitis relies mostly on specific PCR and culturing methods, but commercial syndromic panel assays are bringing a change in diagnostics. With multiplexed analysis, the identification of the pathogen is potentially faster, and less sample material is needed. The novel QIAstat-Dx meningitis/encephalitis panel assay is intended for the rapid identification of pathogens from cerebrospinal fluid for suspected central nervous system (CNS) infection, which is a life-threatening condition and difficult to diagnose. We studied the performance of this panel assay using patient samples and dilution series of selected viruses. The evaluation data for this novel meningitis/encephalitis panel assay are useful for other clinical laboratories and organizations using or considering using this test.

## INTRODUCTION

Central nervous system (CNS) infections are rather rare but are associated with high mortality and morbidity rates. Infectious meningitis, encephalitis, or meningoencephalitis may be caused by bacteria, viruses, or fungi. The most frequent pathogens causing bacterial meningitis worldwide are Streptococcus pneumoniae, Haemophilus influenzae, and Neisseria meningitidis (1–3). Aseptic, or viral, meningitis is often caused by enteroviruses (4, 5). Other viral pathogens include herpesviruses and arboviruses, which also cover the most common viral encephalitis pathogens, including herpes simplex virus 1 (HSV-1), HSV-2, and varicella-zoster virus (VZV) (6). Furthermore, HSV-1 is the most common cause of fatal sporadic encephalitis. HSV encephalitis is an emergency situation with a mortality rate of around 70% if left untreated, and even when treated, the mortality rate is high, and long-term effects may occur (7–9).

Distinguishing different central nervous system infections based only on symptoms is difficult. Therefore, prompt laboratory analysis is needed to identify possible pathogens and guide treatment. Microbiological laboratory analyses from cerebrospinal fluid (CSF) samples traditionally include bacterial culturing, microscopy and antigen tests (10, 11). Viral pathogens are often identified using singleplex real-time PCR methods, which have generally shown high sensitivity and specificity (11, 12). Also, metagenomic sequencing methods have been developed for the identification of CNS infections (13).

To further improve targeted molecular identification, rapid diagnostics have been developed. The aims are to reduce the time of PCR processes by abolishing manual steps, decrease the sample volume needed, and improve diagnostics by combining several PCR tests into one multiplex PCR panel test. There are now two CE-IVD-marked rapid diagnostic products available (in cassette format) for meningitis and/or encephalitis multiplex PCR testing from CSF samples: the BioFire FilmArray meningitis/encephalitis (ME) panel (FA/ME), which has been available since 2015, and the novel QIAstat-Dx meningitis/encephalitis panel (QIA/ME). FA/ME includes 14 viral, bacterial, and fungal pathogens, while QIA/ME includes 15 pathogens (see Table 1 for more details on the panels).

**TABLE 1:** Pathogens detected by the QIAstat-Dx meningitis/encephalitis panel and the BioFire FilmArray meningitis/encephalitis panel and information on the reference methods^*a*^

Pathogen	Detection of pathogen by panel		Reference method(s) (reference)	Template vol (μL)
	QIA/ME	FA/ME		
Viruses^*b*^				
CMV	NA	X	Real-time PCR (31)	10
HSV-1	X	X	Real-time PCR using Qiagen artus HSV-1/2	5
HSV-2	X	X	Real-time PCR using Qiagen artus HSV-1/2	5
Human EV	X	X	Real-time PCR (32) or Cepheid Xpert EV	7
HHV-6	X	X	Real-time PCR (33)	10
HPeV	X	X	Real-time PCR (32)	7
VZV	X	X	Real-time PCR using Qiagen artus VZV	5
Bacteria				
Escherichia coli K1	X	X	Pathogen not tested in this study	
Haemophilus influenzae	X	X	Bacterial culturing	
Listeria monocytogenes	X	X	Bacterial culturing	
Mycoplasma pneumoniae	X	NA	Pathogen not tested in this study	
Neisseria meningitidis	X	X	Bacterial culturing	
Streptococcus agalactiae	X	X	Bacterial culturing	
Streptococcus pneumoniae	X	X	Bacterial culturing	
Streptococcus pyogenes	X	NA	Pathogen not tested in this study	
Fungi				
Cryptococcus gattii/C. neoformans	X	X	Antigen testing, microscopy	

a See references 31–33. QIA/ME, QIAstat-Dx meningitis/encephalatis panel; FA/ME, BioFire FilmArray meningitis/encephalitis panel; CMV, cytomegalovirus; HSV-1, herpes simplex virus 1; EV, enterovirus; HHV-6, human herpesvirus 6; HPeV, human parechovirus; VZV, varicella-zoster virus; NA, not applicable.

b For viruses, PCR was performed using NucliSENS easyMAG with a sample volume of 200 μL and an elution volume of 25 μL.

Multiplex tests have been welcomed with high hopes of reducing the time needed for specific diagnosis, the amount of CSF needed, empirical antibiotic use, and overall costs, etc., and some positive outcomes have already been reported (14–16). Multiplex panel assays have also raised concerns about their use and the interpretation of their results (17, 18), and thus, the evaluation of the new options is of great importance. Comparisons of the two panels will give valuable information on the performances of these assays and practical knowledge for clinical laboratories interpreting the results of panel assays. In this study, the performance of the newly released QIA/ME assay was evaluated by comparing it with the performances of FA/ME and the reference methods used in our laboratory. The study was further continued by focusing specifically on HSV-1, HSV-2, and VZV detection performance by comparing six different commercial assays using dilution series.

## RESULTS

### Results for patient samples.

We analyzed the CSF samples in parallel using QIA/ME and FA/ME. FA/ME was considered a comparator method in this analysis. The positive percent agreement (PPA) and negative percent agreement (NPA) of QIA/ME were 100% for all included pathogens except S. pneumoniae (Table 2). The NPA for S. pneumoniae was 98.7%, and this was due to one sample that was positive by our diagnostic reference method and QIA/ME but negative by FA/ME. Unfortunately, there was not enough sample material for further discrepancy analyses.

**TABLE 2:** Performance of QIA/ME against FA/ME using patient samples^*a*^

Target	No. of samples with result				PPA	95% CI for PPA (%)	NPA	95% CI for NPA (%)
	TP	FP	FN	TN^*b*^				
EV	6	0	0	73	100	55.7 to 100	100	94.0 to 100
HHV-6	1	0	0	78	100	16.8 to 100	100	94.4 to 100
HPeV	4	0	0	75	100	45.4 to 100	100	94.2 to 100
HSV-1	9	0	0	70	100	65.5 to 100	100	93.8 to 100
HSV-2	11	0	0	68	100	70.0 to 100	100	93.6 to 100
VZV	6	0	0	73	100	55.7 to 100	100	94.0 to 100
H. influenzae	2	0	0	77	100	29.0 to 100	100	94.3 to 100
L. monocytogenes	5	0	0	74	100	51.1 to 100	100	94.1 to 100
N. meningitidis	1	0	0	78	100	16.8 to 100	100	94.4 to 100
S. agalactiae	6	0	0	73	100	55.7 to 100	100	94.0 to 100
S. pneumoniae	5	1	0	73	100	51.1 to 100	98.7	92.0 to >99.9
C. gattii/C. neoformans	1	0	0	78	100	16.8 to 100	100	94.4 to 100

Total	57	1	0	16	100	92.5 to 100	94.1	71.1 to >99.9

a TP, true positive; FP, false positive; FN, false negative; TN, true negative; PPA, positive percent agreement; CI, confidence interval; NPA, negative percent agreement.

b In total, 16 negative samples were run. The true-negative count is the sum of the 16 negative samples plus the number of all other samples run by FA/ME (63 in total) with different targets detected (for example, the true-negative count for enterovirus was calculated as 63 − 6 + 16 = 73).

The performances of the QIA/ME and FA/ME tests were similar when positive results were compared to the results of the diagnostic reference methods (see Tables 4 and 5). The overall PPAs were 95.1% for QIA/ME and 93.8% for FA/ME. The lowest single PPA (50%) was seen for human herpesvirus 6 (HHV-6) (both QIA/ME and FA/ME versus the reference method); however, there were only two positive samples included in the evaluation. One sample was weakly positive by the reference method (quantification cycle [*C_q_*] value of 39.3), but both QIA/ME and FA/ME gave a negative test result, which was deemed a false-negative result. The other HHV-6-positive sample was a doubly positive sample with additional cytomegalovirus (CMV) positivity. This was correctly identified as doubly positive by FA/ME, but QIA/ME lacks CMV identification.

The diluted samples (Table 3) performed well: all diluted bacterial samples gave the expected results according to the reference method. Two diluted samples, one positive for HSV-1 and another positive for HSV-2, were negative by both QIA/ME and FA/ME. We reanalyzed these samples using the reference method, which identified HSV-1 (*C_q_* value of 28.1) and HSV-2 (*C_q_* value of 33.2) respectively. The unidentified HSV samples decreased the PPAs to 90% for HSV-1 and 91.7% for HSV-2 (Tables 4 and 5).

**TABLE 3:** Results for positive patient samples using the reference method, QIA/ME, and FA/ME^*a*^

Reference method interpretation(s)	Reference method *C_q_* value(s) or growth on plate	Interpretation of QIA/ME result	QIA/ME *C_q_* value	Interpretation(s) of FA/ME result	Dilution factor if diluted
CMV	24.1	Negative	NA	CMV	
EV	26.1	EV	31.9	EV	1:2
EV	29.4	EV	32.3	EV	
EV	29.5	EV	35.3	EV	
EV	29.9	EV	34.9	EV	1:2
EV	30.3	EV	34.6	EV	
EV	33.8	EV	28.8	EV	1:3
HHV-6	39.3 (rerun, 37.7)	Negative	NA	Negative	
HHV-6 and CMV	HHV-6, 32.8; CMV, 38.9	HHV-6	29.7	CMV and HHV-6	
HPeV	31.9	HPeV	33.2	HPeV	1:4
HPeV	32.1	HPeV	34.5	HPeV	1:3
HPeV	34.2	HPeV	36.9	HPeV	
HPeV	36.6	HPeV	35.1	HPeV	
HSV-1	13.4	HSV-1	22.6	HSV-1	
HSV-1	20.1	HSV-1	31.1	HSV-1	
HSV-1	20.5	HSV-1	30.8	HSV-1	
HSV-1	21.0	Error (rerun, HSV-1)	31.1	HSV-1	
HSV-1	23.5	HSV-1	31.0	HSV-1	
HSV-1	23.8	Error (rerun, HSV-1)	31.0	HSV-1	
HSV-1	24.0	Failed (rerun, HSV-1)	33.3	HSV-1	
HSV-1	24.6	HSV-1	32.0	HSV-1	
HSV-1	26.5	Error (rerun, HSV-1)	33.9	HSV-1	
HSV-1	28.0 (rerun, 28.1)	Negative	NA	Negative	1:3
HSV-2	23.6	HSV-2	28.8	HSV-2	
HSV-2	24.0	HSV-2	32.3	HSV-2	
HSV-2	25.1	HSV-2	32.9	HSV-2	
HSV-2	25.4	HSV-2	33.4	HSV-2	
HSV-2	26.1	HSV-2	31.9	HSV-2	
HSV-2	26.8	HSV-2	34.0	HSV-2	
HSV-2	26.9	HSV-2	36.4	HSV-2	
HSV-2	27.5	HSV-2	35.6	HSV-2	
HSV-2	28.5 (rerun, 33.2)	Negative	NA	Negative	1:3
HSV-2	28.9	HSV-2	34.6	HSV-2	
HSV-2	29.4	HSV-2	34.9	HSV-2	
HSV-2	29.5	HSV-2	37.1	HSV-2	
VZV	20.6	VZV	26.7	VZV	
VZV	24.0	VZV	31.1	VZV	
VZV	26.35	VZV	30.1	VZV	
VZV	26.8	VZV	33.3	VZV	
VZV	27.9	VZV	32.5	VZV	
VZV	32.2	VZV	35.5	VZV	
H. influenzae	+++^*b*^	H. influenzae	22.1	H. influenzae	1:2.5
H. influenzae	+++	H. influenzae	26.9	H. influenzae	
H. influenzae	+	Failed	NA	H. influenzae	1:4
L. monocytogenes	+++	L. monocytogenes	23.6	L. monocytogenes	
L. monocytogenes	++	L. monocytogenes	34.4	L. monocytogenes	1:3
L. monocytogenes	++	L. monocytogenes	35.2	L. monocytogenes	1:10
L. monocytogenes	++	L. monocytogenes	35.5	L. monocytogenes	
L. monocytogenes	++	L. monocytogenes	32.4	L. monocytogenes	1:1.75
N. meningitidis (type B)	++	N. meningitidis	17.7	N. meningitidis	1:1.25
S. agalactiae	+++	S. agalactiae	23.2	S. agalactiae	1:2
S. agalactiae	++	S. agalactiae	23.6	S. agalactiae	1:1.25
S. agalactiae	++	S. agalactiae	28.4	S. agalactiae	1:1.4
S. agalactiae	++	S. agalactiae	24.2	S. agalactiae	1:10
S. agalactiae	+	S. agalactiae	29.4	S. agalactiae	
S. agalactiae	+	S. agalactiae	33.3	S. agalactiae	1:10
S. pneumoniae	+	S. pneumoniae	29.0	S. pneumoniae	1:5
S. pneumoniae	+++	S. pneumoniae	16.6	S. pneumoniae	1:2
S. pneumoniae	+++	S. pneumoniae	15.4	S. pneumoniae	
S. pneumoniae	+++	S. pneumoniae	16.9	Negative	
S. pneumoniae	+++	S. pneumoniae	22.1	S. pneumoniae	1:2.5
S. pneumoniae	NA	S. pneumoniae	22.8	S. pneumoniae	1:10
C. neoformans	NA	C. gattii/C. neoformans	29.9	C. gattii/C. neoformans	1:10

a The *C_q_* results from the discrepancy analysis or rerun interpretations after errors or failed results are shown in parentheses (reruns). The dilution factor is presented if applicable. NA, not applicable.

b Growth on plate: +, single colonies; ++, multiple segments; +++, confluent growth.

**TABLE 4:** Performance of QIA/ME against the reference method using positive patient samples^*a*^

Target	No. of samples with result by QIA/ME		PPA	95% CI (%)
	TP	FN		
EV	6	0	100	55.7 to 100
HHV-6	1	1	50	9.5 to 90.5
HPeV	4	0	100	45.4 to 100
HSV-1	9	1	90	57.4 to >99.9
HSV-2	11	1	91.7	62.5 to >99.9
VZV	6	0	100	55.7 to 100
H. influenzae	2	0	100	29.0 to 100
L. monocytogenes	5	0	100	51.1 to 100
N. meningitidis	1	0	100	16.8 to 100
S. agalactiae	6	0	100	55.7 to 100
S. pneumoniae	6	0	100	55.7 to 100
C. gattii/C. neoformans	1	0	100	16.8 to 100
Total	58	3	95.1	86.0 to 98.9

a TP, true positive; FN, false negative; PPA, positive percent agreement; CI, confidence interval.

**TABLE 5:** Performance of FA/ME against reference method using positive patient samples^*a*^

Target	No. of samples with result by FA/ME		PPA	95% CI (%)
	TP	FN		
CMV	2	0	100	29.0 to 100
EV	6	0	100	55.7 to 100
HHV-6	1	1	50.0	9.5 to 90.5
HPeV	4	0	100	45.4 to 100
HSV-1	9	1	90.0	57.4 to >99.9
HSV-2	11	1	91.7	62.5 to >99.9
VZV	6	0	100	55.7 to 100
H. influenzae	3	0	100	38.3 to 100
L. monocytogenes	5	0	100	51.1 to 100
N. meningitidis	1	0	100	16.8 to 100
S. agalactiae	6	0	100	55.7 to 100
S. pneumoniae	5	1	83.3	41.8 to 98.9
C. gattii/C. neoformans	1	0	100	16.8 to 100
Total	60	4	93.8	84.6 to 98.0

a TP, true positive; FN, false negative; PPA, positive percent agreement; CI, confidence interval.

### Results of herpesvirus dilutions.

To demonstrate the performance of the QIA/ME assay near the limit of detection, we prepared a dilution series of HSV-1, HSV-2, and VZV (Table 6). As expected, less-multiplexed real-time PCR methods with separate extraction (especially RealStar assays) detected much more dilute samples than the panel assays, and the viral target was detected even in the most dilute samples. Overall, FA/ME performed better than QIA/ME with herpesvirus dilutions. FA/ME seemed to perform even better than the Simplexa HSV 1&2 direct assay, especially with HSV-2 dilutions (Table 6). The Simplexa HSV 1&2 direct assay targets only HSV-1 and -2 and is also considered a rapid diagnostic assay. Allplex Meningitis-V1 is a multiplex assay but with a separate extraction step, such as in the other two real-time PCR methods. This assay also performed well, detecting only slightly fewer positives than artus HSV-1/2 and RealStar HSV with HSV-1 and -2.

**TABLE 6:** Results for the HSV-1, HSV-2, and VZV dilution series analyzed using six different assays^*a*^

Dilution and parameter	Value for assay					
	QIA/ME	FA/ME	Simplexa HSV 1&2 direct	Allplex Meningitis-V1	artus HSV-1/2	RealStar HSV
HSV-1						
1:100,000						
Result	Positive	Positive	Positive	Positive	Positive	Positive
*C_q_* or qualitative value	36.0	+	35.7	33.9	21.9	34.3
1:200,000						
Result	Positive	Positive	Positive	Positive	Positive	Positive
*C_q_* for replicate 1/replicate 2 or qualitative value	36.2/36.4	+/+	37/38.5	35.4/34.5	22.9/24.5	35.5/35.0
1:1,000,000						
Result	Negative	Borderline	Borderline	Positive	Positive	Positive
*C_q_* for replicate 1/replicate 2/replicate 3 or qualitative value	0/0	+/−	0/37.9/0	37.7/37.6/37.5	24.5/27.5/26.3	37.7/36.8/36.3
1:2,000,000						
Result	NA	Positive	Borderline	Positive	Positive	Positive
*C_q_* for replicate 1/replicate 2/replicate 3 or qualitative value		+/+	0/39.1/0	38.8/0/27.9	0/27.4/27.7	36.8/36.9/37.5
1:10,000,000						
Result	NA	Borderline	Negative	Negative	Borderline	Borderline
*C_q_* for replicate 1/replicate 2/replicate 3 or qualitative value		+/−	0/0/0	0/0/0	0/27.8/0	0/38.6/0

HSV-2						
1:100,000						
Result	Positive	Positive	Positive	Positive	Positive	Positive
*C_q_* or qualitative value	35.6	+	35.3	33.7	24.7	34.7
1:200,000						
Result	Positive	Positive	Positive	Positive	Positive	Positive
*C_q_* for replicate 1/replicate 2 or qualitative value	36.0/35.9	+/+	35.7/38.2	37.3/34.8	24.9/26.5	35.5/35.1
1:1,000,000						
Result	Borderline	Positive	Borderline	Borderline	Positive	Positive
*C_q_* for replicate 1/replicate 2/replicate 3 or qualitative value	38.0/0	+/+	0/38.4/0	0/36.7/0	0/29.0/27.7	37.1/38.0/37.0
1:2,000,000						
Result	Negative	Positive	Negative	Positive	Borderline	Positive
*C_q_* for replicate 1/replicate 2/replicate 3 or qualitative value	0/0	+/+	0/0/0	0/36.1/37.3	0/30.5/0	40.20/43.2/38.3
1:10,000,000						
Result	NA	Borderline	Negative	Negative	Negative	Positive
*C_q_* for replicate 1/replicate 2/replicate 3 or qualitative value		+/−	0/0/0	0/0/0	0/0/0	0/41.7/39.9

VZV						
1:100,000						
Result	Positive	Positive	Failed^*b*^	Positive	Positive	Positive
*C_q_* or qualitative value	33.7	+	NA	31.4	27.3	28.9
1:200,000						
Result	Positive	Positive	Positive	Positive	Positive	Positive
*C_q_* for replicate 1/replicate 2 qualitative value	36.9	+	38.2/37.3	32.6/33.8	28.3/31.6	29.9/30.5
1:1,000,000						
Result	Positive	Positive	Positive	Positive	Positive	Positive
*C_q_* for replicate 1/replicate 2/replicate 3 or qualitative value	37.3/37.1	+/+	38.9/37.9/39.9	34.6/38.6/35.8	30.3/34.9/32.3	31.9/32.9/32.3
1:2,000,000						
Result	Borderline	Positive	Positive	Positive	Positive	Positive
*C_q_* for replicate 1/replicate 2/replicate 3 or qualitative value	38.7/0	+/+	38.9/40.1/0	0/37.1/37.5	33.1/38.4/33.7	33.2/33.9/33.7
1:10,000,000						
Result	Negative	Borderline	Negative	Borderline	Negative	Positive
*C_q_* for replicate 1/replicate 2/replicate 3 or qualitative value	0	+/−	0/0/0	0/37.7/0	0/0/0	35.8/36.7/37.7

a Results are interpreted as positive, negative, or borderline. The numbers below the results are *C_q_* values obtained from replicate analyses (1 to 3 replicates were run for each dilution). The BioFire FilmArray ME (FA/ME) assay does not give *C_q_* values. Dark shading indicates a positive result. Light shading indicates results that are in the detection limit area and where the result was positive in only one replicate. QIA/ME, QIAstat-Dx ME panel.

b Invalid internal control result. The assay does not make the interpretation. The sample was not rerun.

*C_q_* values are shown in Table 6. However, *C_q_* values cannot be used for quantitation for samples in qualitative assays, but the values are a tool to estimate if the sample is near the limit of detection or strongly positive.

### Results for external quality control samples.

QCMD (Quality Control for Molecular Diagnostics) 2021 central nervous system I (viral meningitis and encephalitis) external quality assessment (EQA) proficiency samples were used to evaluate the performances of the QIA/ME and FA/ME tests for viral targets (HSV, VZV, enterovirus, and parechovirus). Both tests performed well (Table 7) and had 100% consistency with the expected results.

**TABLE 7:** Results for quality control samples run using QIA/ME and FA/ME (QCMD 2021 central nervous system I [viral meningitis and encephalitis] EQA program)

QC sample code	QC sample content	Results of QIA/ME	Results of FA/ME
CNSI21S-01	EV (echovirus 30)	EV	EV
CNSI21S-02	HSV-2	HSV-2	HSV-2
CNSI21S-03	HPeV (type 1)	HPeV	HPeV
CNSI21S-04	VZV (9/84)	VZV	VZV
CNSI21S-05	Negative	Negative	Negative
CNSI21S-06	HSV-1	HSV-1	HSV-1
CNSI21S-07	EV (A71)	EV	EV
CNSI21S-08	HPeV (type 3)	HPeV	HPeV
CNSI21S-09	HSV-1	HSV-1	HSV-1
CNSI21S-10	VZV (Ellen)	VZV	VZV
Concordance (%)		100	100

### User experience and device functionality of QIA/ME.

Rapid diagnostics have deliberately been designed to be user-friendly, and QIA/ME is not an exception: the handling of the assay cassette is even faster and easier than with FA/ME as the user pipettes the sample directly into the cassette, which is then loaded into the QIAstat-Dx analyzer. Using the QIAstat-Dx analyzer was as easy as using the FilmArray analyzer. The user does not need any extra programs or computers as the results can be read directly from the analyzer’s touch screen, or the results can be sent to the laboratory information management system (LIS) (not tested in this study). Clear advantages of QIA/ME are the reported *C_q_* values and PCR amplification curves.

The total error rate of QIA/ME in this study was 6.5% (3 errors and 4 failed results from 108 samples run, including dilutions and quality control [QC] samples). The FA/ME error rate was 0% (total of 115 samples run). There were three errors with QIA/ME during the study; two of them were due to QIAstat-Dx analyzer problems. One error was due to a test cassette problem. These three samples were rerun in another analyzer and other test cassettes, and they received the expected results in the second run (all three were positive for HSV-1). In addition, four patient samples gave failed results with QIA/ME. Three of these samples were rerun and received the same results as those of the FA/ME and reference methods (two negative samples and one HSV-1-positive sample). The fourth failed sample was H. influenzae positive by the reference method and FA/ME. Unfortunately, there was not enough CSF left to rerun this sample with QIA/ME.

## DISCUSSION

Infectious meningitis, encephalitis, or meningoencephalitis is always a severe and life-threating condition. There are many possible causes, including bacterial, viral, or fungal agents. The rapid identification of the infectious agent is essential for the selection of the appropriate treatment, and multiplex panel assays aim to answer this need. In this study, we evaluated the recently released CE-IVD-marked QIAstat-Dx meningitis/encephalitis panel (QIA/ME). The results of the patient sample analyses indicated that the new QIA/ME assay and the BioFire FilmArray meningitis/encephalitis (FA/ME) assay are comparable and perform quite similarly.

In this study, with a relatively small sample size, the positive and negative percent agreements of QIA/ME and FA/ME (Tables 3 to 5) were slightly better than the specificity and sensitivity values seen in previous studies evaluating FA/ME (17–24). The PPA for QIA/ME (compared to the reference methods) was 95% in our study, and for example, Tansarli and Chapin (18) estimated a 90% mean sensitivity (and a 97% mean specificity) for FA/ME in their meta-analysis.

Since CNS infection is a severe condition with considerable mortality and morbidity, there should not be false-positive or -negative results by the assays. However, previous studies have drawn attention to false-positive and -negative results using FA/ME (17, 18). In our study, no false-positive results were observed, but a few false-negative results were detected. Previous reports have found false-negative results mostly for HSV-1, HSV-2, enteroviruses, and Cryptococcus gattii/C. neoformans using FA/ME (17, 18, 22). In this study, QIA/ME was unable to identify HSV-1-, HSV-2-, and HHV-6-positive samples (one each). These same samples were also negative by FA/ME, which may suggest similar problems with the identification of herpesviruses. In addition, we noted one false-negative result for S. pneumoniae using FA/ME. However, S. pneumoniae has previously been linked mainly to false-positive results (18, 19, 23).

Further studies using dilution series suggested that FA/ME might be more sensitive than QIA/ME for the detection of herpes simplex viruses. There was a clear difference in the results of HSV-1 and HSV-2 dilution series by QIA/ME and FA/ME (Table 6). Yet this experiment was prepared with only one representative strain, and the abilities of the assays to detect different strains may vary. The dilution series experiment was prompted by previous reports on the lack of sensitivity of the FA/ME assay for the detection of herpesviruses (12, 13, 17; L. Mannonen, R. Loginov, T. Holma, J. Antikainen, unpublished data).

In the dilution series experiment, we tested other commercial assays in addition to QIA/ME and FA/ME. For instance, we used the multiplexed Allplex Meningitis-V1 assay from Seegene. To our knowledge, there are no such previous reports on the Allplex Meningitis-V1 panel assay. Overall, one or two target PCR methods are still probably the most common methods used to detect herpesviruses (12). artus from Qiagen and RealStar from Altona were included in the evaluation of the dilution series of HSV-1, HSV-2, and VZV, and they performed better than the multiple-target PCR panel assays, as expected. The fewest differences between the assays were seen for VZV detection.

Published data are accumulating for FA/ME, making it clear that it cannot be used as a stand-alone test. Also, both FA/ME and QIA/ME have several limitations listed in their instructions for use (BioFire FilmArray ME panel CE-IVD instruction booklet [BioFire Diagnostics, bioMérieux LLC] and QIAstat-Dx ME panel instructions for use [handbook], January 2022 [Qiagen GmbH]). One of the limitations is the inability to identify all viral or bacterial strains with sufficient sensitivity. For instance, neither of the tests identifies all HSV-1 strains, and the sensitivity for HSV-1, which is one of the most important pathogens, is compromised. Furthermore, it should be noted that the analytical verification of QIA/ME has shown a potential risk of false-negative results for HSV-1 when S. pneumoniae is also present in the sample. Previous studies have suggested that precautions should be taken when diagnosing a patient based solely on FA/ME results and that additional confirmatory testing is still needed (17, 23, 24). These studies suggest the need for confirmatory testing, especially for HSV-1, HSV-2, and enteroviruses. The results of our study also support the need for further confirmatory testing for at least HSV-1 and HSV-2 when using QIA/ME. This finding naturally needs further studies of QIA/ME with a wider range of samples.

However, rapid methods have great potential in the diagnostics of meningitis and/or encephalitis, and these assays are likely most useful in emergency departments. There are reports of clearly positive impacts of the use of panel assays for meningitis and/or encephalitis diagnostics, for instance, in reducing the time to diagnosis and shortening empirical antibiotic use (14, 15). QIA/ME is also a rapid assay with a run time of 1 h and with the availability of an LIS for fast and reliable reporting to the clinic. Both QIA/ME and FA/ME need only 200 μL of a CSF sample, which is important with the usually low sample volume. However, in cases of an error or a failed result, the laboratory must rerun the sample, and more sample is then needed. This makes the reliability of the assays and analyzers critical. In this study, FA/ME was more reliable than QIA/ME, with 0% and 6.5% error rates, respectively.

Interestingly, some laboratories have also reported an increase in the prevalence of findings when using the FA/ME test (25). As a fair number of meningitis or encephalitis patients are left without a final diagnosis of the causative agent, it might be beneficial to test more patients with rapid molecular assays, but it may also add some false-positive results. For instance, the instructions for use for QIA/ME warn that Cutibacterium acnes or Mycoplasma genitalium may give a false-positive Mycoplasma pneumoniae result, and similarly, Haemophilus haemolyticus may be falsely identified as Haemophilus influenzae. Also, not all findings may be equally significant in the panel assays, and it is of the utmost importance to consider all aspects of the patient’s situation when making clinical decisions. Although HHV-6 can cause severe CNS disease, especially in immunocompromised hosts, a positive HHV-6 finding may also result from subclinical reactivation of the latent virus or chromosomal integration and may not be relevant for the CNS infection (26–29). Furthermore, one can detect only those pathogens that are involved in the panel and not potential new or rare causes, which could be found, for example, by bacterial culture. Thus, rapid diagnostics cannot replace all of the current methods since they may have insufficient sensitivity, and the panels lack many pathogens that potentially cause meningitis or encephalitis. Moreover, bacterial antibiotic sensitivity cannot be fully determined by rapid diagnostic methods.

Ideally, rapid testing for the most common microbes causing CNS infections simultaneously improves patient care and outcomes and reduces the overall costs of patient treatment. However, the most important thing is the quality of the test. Here, we have shown that there is still room for improvement in CSF diagnostics using rapid cassette tests. Highly multiplexed assays are usually more difficult to design so that the sensitivity for all targets would be optimal. Therefore, more targeted, less multiplexed panels in a rapid cassette format for the diagnosis of CNS infections might make sense.

### Study limitations.

This is a retrospective single-center study in which frozen residual CSF samples from clinical diagnostics were used. Testing of CNS infection assays are usually limited by the small sample size, and the samples are often prechosen based on availability, which was also true for this study. We aimed to evaluate the performance of QIA/ME with as wide of a range of positive CSF samples as possible, and this study does not represent the real-life situation in a clinical laboratory, where the majority of CSF samples are negative. Based on the availability of residual samples, this study lacked some bacterial pathogen targets completely, and some pathogen targets had only a few representatives. With the preselected samples and with the priority of having enough sample material, this study had a rather narrow-range *C_q_* value sample group, which does not reflect the real clinical situation (Table 3). Thus, the material lacked more challenging samples with low viral loads.

### Conclusions.

The recently established QIA/ME assay is a rapid diagnostic multiplex PCR assay that significantly reduces the time to a result and the amount of a CSF sample needed when meningitis or encephalitis is suspected. QIA/ME performed very similarly to FA/ME, and further studies are expected to confirm and widen these first observations of the performance of this assay. Panel assays such as QIA/ME and FA/ME provide an option for the rapid diagnostics of central nervous system infections in clinical laboratories, but users should interpret the results carefully in the context of individual patients.

## MATERIALS AND METHODS

### Panel assays and reference methods.

The QIAstat-Dx meningitis/encephalitis (ME) panel (QIA/ME; Qiagen GmbH, Hilden, Germany) was used according to the manufacturer’s instructions with QIAstat-Dx analyzer 1.0 (Qiagen GmbH) and QIAstat-Dx Application software 1.4.0.

The BioFire FilmArray meningitis/encephalitis (ME) panel (FA/ME; BioFire Diagnostics, bioMérieux LLC, Salt Lake City, UT, USA) was used according to the manufacturer’s instructions with the BioFire FilmArray Torch instrument and software (BioFire Diagnostics, bioMérieux LLC). All of the pathogens included in QIA/ME and FA/ME and the reference methods used (PCR methods) are listed in Table 1. For culturing, the clinical samples were cultured at the Helsinki University Hospital Laboratory using standard methods (30).

### Samples and patients.

Patient samples used in this study were residual frozen CSF samples sent to the Helsinki University Hospital Laboratory from 2017 to 2022. The samples were sent for meningitis and/or encephalitis diagnostics. The main selection criterion was that there was enough residual CSF to carry out both the QIA/ME and FA/ME tests (200 μL is required for each analysis). Second, we aimed to have representative samples for most of the pathogens included in the two panels (see Table 1 for the list of pathogens). We did not have samples for Escherichia coli K1, Streptococcus pyogenes (only for QIA/ME), and Mycoplasma pneumoniae (only for QIA/ME). The number of samples in each pathogen category was limited by the sample volume. Thus, the number of positive samples was increased by diluting positive patient samples in residual negative CSF. Altogether, 23 samples were diluted, most of which were bacterial samples (dilution factors are shown in Table 3). The residual negative CSF samples were defined negative using the FA/ME assay; in addition, the diluted samples were reanalyzed using the appropriate reference method.

In total, 63 positive CSF samples were analyzed (positive by the reference method). The median age of the patients was 47 years, with a range from a few weeks to 91 years. A total of 57% (*n* = 36) of the positive samples were from male patients, and 43% (*n* = 27) were from female patients. In addition, 16 clinical samples that were negative by FA/ME were analyzed. These negative samples were used for the calculation of the negative percent agreement of QIA/ME versus FA/ME. Besides these negative samples, samples with a positive result for one target (or two targets in the case of a doubly positive result) in the panel were considered negative for all other targets in the statistical calculations.

### Dilution series.

Dilution series of HSV-1, HSV-2, and VZV were tested in parallel with six different PCR methods. The dilution series were prepared from leftover cutaneous samples by diluting these into residual negative CSF (defined as negative by FA/ME). A dilution series of 1:10^2^ to 1:10^7^ was constructed for each virus. The dilution series were analyzed using QIA/ME and FA/ME. In addition, we used Simplexa HSV 1&2 direct (DiaSorin Molecular LLC, Cypress, CA, USA) and Simplexa VZV direct (DiaSorin Molecular LLC) assays with the Liaison MDX instrument according to the manufacturer’s instructions. Dilution series were also analyzed using the Allplex Meningitis-V1 assay (Seegene Inc., Seoul, Republic of Korea), the artus HSV-1/2 LC PCR and VZV LC PCR kits (Qiagen GmbH), and RealStar HSV PCR kit 1.0 and VZV PCR kit 1.0 (Altona Diagnostics GmbH, Hamburg, Germany). For these tests, DNA extraction was performed by using the automated NucliSENS easyMAG system (bioMérieux, Boxtel, Netherlands) with a 200-μL starting sample volume, using a generic protocol, and eluting the sample into 25 μL. The Allplex Meningitis-V1 assay was used with the CFX96 Dx system (Bio-Rad Laboratories, Hercules, CA, USA) and Seegene Viewer (V3.24.00) analysis software. The results of the artus assays were analyzed using the LightCycler 2.0 system (Roche, Basel, Switzerland), and the results of the RealStar assays were analyzed using the Applied Biosystems 7500 real-time PCR system (ABI7500; Thermo Fisher Scientific, Waltham, MA, USA).

### External quality control samples.

The QCMD (Quality Control for Molecular Diagnostics) 2021 central nervous system I (viral meningitis and encephalitis) EQA program was purchased from QCMD (Glasgow, Scotland, UK).

### Statistical calculations.

Positive percent agreement (PPA) was calculated using the equation 100% × TP/(TP + FN), where TP is a true-positive sample and FN is a false-negative sample. Negative percent agreement (NPA)was calculated using the equation 100% × TN/(TN + FP), where TN is a true-negative sample and FP is a false-positive sample.

Confidence intervals were calculated using an online version of GraphPad software (Dotmatics [https://www.graphpad.com/quickcalcs/confInterval1/ {accessed 28 July 2022}]). The modified Wald method was used for all confidence interval calculations.

### Ethics statement.

No ethics evaluation was needed. The study was approved by the Independent Institutional Review Board of the Hospital District of Helsinki and Uusimaa (15 March 2022; HUSLAB 15§/2022, 151/2022).
